# Asiatic acid remodels the gastric precancerous immune microenvironment by targeting STING

**DOI:** 10.3389/fimmu.2026.1896372

**Published:** 2026-08-20

**Authors:** Haotian Li, Li Shen, Jiabao Liu, Xiaolan Su, Man Meng, Jianqin Yang, Runhua Chen, Chen Yang, Yanjun Liu

**Affiliations:** 1 Institute of Basic Theory of Traditional Chinese Medicine, China Academy of Chinese Medical Sciences, Beijing, China; 2 Wangjing Hospital, China Academy of Chinese Medical Sciences, Beijing, China; 3 Department of Digestive Disorders, Beijing Key Laboratory of Diagnosis and Treatment of Functional Gastrointestinal Diseases of Traditional Chinese Medicine, Wangjing Hospital, China Academy of Chinese Medical Sciences, Beijing, China; 4 Beijing University of Chinese Medicine, Beijing, China; 5 Department of Gastroenterology, Dongfang Hospital, Beijing University of Chinese Medicine, Beijing, China; 6 Emergency General Hospital, Beijing, China

**Keywords:** asiatic acid, gastric precancerous lesions, gut microbiota, immune microenvironment, inflammation-to-cancer transformation, STING

## Abstract

**Introduction:**

Chronic inflammation-driven gastric precancerous lesions (GPL) are characterized by progressive immune remodeling toward an immunosuppressive state, yet effective chemopreventive agents targeting this process remain scarce. Here, we identify asiatic acid (AA), a natural pentacyclic triterpenoid, as a STING‑binding compound that contributes to reshaping the gastric immune microenvironment and interfering with the Correa cascade.

**Methods:**

Using a rat model of MNNG‑induced GPL (atrophy, intestinal metaplasia, dysplasia) and MNNG‑treated GES‑1 cells, we assessed histopathological damage, systemic pro‑inflammatory cytokines (TNF, IL‑6, MCP1), and gastric physiological function. STING binding was evaluated by surface plasmon resonance (SPR) with KD measurement and 100 ns molecular dynamics simulations. The cGAS–STING–TBK1–IRF3 axis was examined *in vivo* and *in vitro*. Gut microbiota was analyzed by 16S rRNA sequencing, and the Nrf2–Keap1 antioxidant pathway, mitochondrial membrane potential, and oxidative stress were measured. Rescue experiments were performed using the STING agonist cGAMP.

**Results:**

AA ameliorated histopathological damage, exerted regulatory effects on systemic pro‑inflammatory cytokines with a non‑linear dose‑response pattern, and improved gastric physiological function. Mechanistically, AA directly bound to STING with a KD of 4.0 μM and maintained a stable conformation in simulations, suppressing aberrant activation of the cGAS–STING–TBK1–IRF3 axis. AA also reversed gut microbiota dysbiosis (notably reducing pro‑inflammatory Erysipelotrichales), restored the Nrf2–Keap1 pathway, improved mitochondrial membrane potential, and reduced oxidative stress. Rescue experiments with cGAMP partially abolished AA’s protective effects, supporting the functional involvement of STING signaling in AA’s actions.

**Discussion:**

Collectively, AA interrupts the vicious “microbiota–oxidative stress–STING” loop in a STING‑dependent manner and contributes to remodeling the precancerous immune microenvironment, positioning it as a promising chemopreventive candidate targeting the earliest phase of gastric carcinogenesis.

## 1 Introduction

Gastric carcinogenesis is a multistep sequential process that follows the classic Correa cascade (1). A combination of factors, such as environmental pollution and oxidative stress, is contributing to a gradual increase in the incidence of cancer (2–4). In this protracted chain of inflammation-to-cancer transformation, the stages spanning atrophy, intestinal metaplasia, and dysplasia represent not merely morphological alterations of epithelial cells, but more importantly, a critical turning point—where the local gastric microenvironment, under persistent inflammatory stimuli, gradually shifts from a simple inflammatory response toward an immunosuppressive state (5), providing fertile soil for dysplastic cells to evade immune surveillance and eventually progress to gastric cancer. From a tumor immunology perspective, this spatial immune remodeling occurring during the gastric mucosal precancerous stage is inherently highly parallel to the mechanisms underlying tumor microenvironment formation (6). Hence, in-depth elucidation of the molecular switches that drive immune remodeling in this gastric precancerous microenvironment, and the identification of interventions capable of blocking the inflammation-to-cancer conversion at this reversible stage, hold profound scientific significance for the chemoprevention of gastric cancer.

The cGAS-STING signaling pathway is a central hub that senses cytosolic double-stranded DNA and bridges innate and adaptive immunity (7). Some studies have shown that oxidative stress accompanied by chronic inflammation can lead to mitochondrial damage, and mitochondrial DNA leakage into the cytoplasm can continuously activate STING, triggering cascade activation of downstream TBK1-IRF3 and NF-κB signaling (8, 9). This drives massive release of inflammatory cytokines such as TNF and IL-6, which not only sustains the local chronic ‘inflammatory’ environment but, more importantly, promotes the formation and maintenance of an immunosuppressive microenvironment by upregulating PD-L1 expression and recruiting immunosuppressive cells, thereby propelling the transition from “inflammation” to “cancer.” (10) Of particular note, through integrated metagenomic and metabolomic analyses, our study revealed that in the gastric precancerous state, oxidative phosphorylation pathways are significantly enriched in the gut microbiota, while the antioxidant metabolic network centered on glutathione is severely disrupted. These multi-omics clues strongly suggest that hyperactive oxidative phosphorylation in the microbiota may exacerbate the overall oxidative burden of the host, leading to excessive consumption of glutathione and collapse of the antioxidant defense, which in turn further aggravates mitochondrial damage and cytosolic DNA leakage, forming a vicious positive-feedback loop of “microbiota metabolic dysregulation–oxidative stress–persistent STING activation.” Concurrently, this vicious cycle is tightly intertwined with dysregulation of the intracellular Keap1-Nrf2/p62 antioxidant axis, together constituting the molecular basis that drives the remodeling of the gastric mucosal immune microenvironment from immune surveillance toward immunosuppression. Therefore, STING emerges as a highly promising integrative target for intervening in the remodeling of the gastric precancerous microenvironment and blocking the inflammation-to-cancer transformation at its source.

Asiatic acid (AA), a pentacyclic triterpenoid natural product derived from the traditional medicinal herb Centella asiatica, possesses well-established anti-inflammatory, antioxidant, and multi-organ protective effects (11–13). However, whether it can reprogram the immune microenvironment at the gastric precancerous stage by modulating the cGAS-STING signaling axis, thereby halting the inflammation-to-cancer transformation, has remained unexplored (14, 15). The data-driven research paradigm is becoming increasingly important (16, 17). On this basis, using molecular docking, surface plasmon resonance, and molecular dynamics simulations, our study provides the first evidence that AA can directly bind to and inhibit the STING protein. We further employed a rat model of gastric precancerous lesions (presenting gastric mucosal atrophy, intestinal metaplasia, and dysplasia) induced by MNNG combined with multiple factors, and systematically evaluated the ameliorative effects of AA on the gastric mucosal inflammatory cytokine network, oxidative stress injury, and gut microbiota dysbiosis. Focusing on STING and its downstream Keap1-Nrf2/p62 signaling axis, we demonstrated that by targeting STING, AA breaks the vicious “microbiota–oxidative stress–STING activation” circuit, thereby effectively remodeling the gastric immune microenvironment at the precancerous stage and blocking its further progression toward dysplasia and gastric cancer (18). This study aims to provide novel scientific evidence for developing asiatic acid as a chemopreventive drug candidate capable of intervening in the formation of the gastric cancer immune microenvironment at its source.

## 2 Methods

### 2.1 Animal model of gastric precancerous lesions

Male SD rats (6 weeks old) were randomly assigned. GPL was induced using a multi−factorial protocol (19, 20): 120 μg/mL MNNG in drinking water (ad libitum, amber bottles), combined with 2% sodium salicylate gavage (5 mL/kg, twice weekly) and an irregular feeding schedule (2 days feeding/1 day fasting). The induction lasted 32 weeks, and successful GPL (atrophy, intestinal metaplasia, dysplasia) was confirmed histologically at weeks 24, 28, and 32.

### 2.2 Experimental design and AA treatment

At week 32, rats were divided into five groups (n = 10 each): normal control, model (vehicle, 0.5% CMC−Na), and AA low− (12.5 mg/kg), medium− (25 mg/kg), and high−dose (50 mg/kg) groups, which dosages were referenced from the literature (21). AA (Solarbio, IA1010) was freshly suspended in 0.5% CMC−Na and administered daily by oral gavage for 8 consecutive weeks. Body weight and food/water intake were recorded weekly. After the final treatment, rats were fasted for 24 h, anesthetized, and gastric tissues, blood, and gastric juice were collected.

### 2.3 Histopathological evaluation

Gastric tissues were fixed in 4% paraformaldehyde, paraffin−embedded, sectioned (4–6 μm), and stained with H&E. Histological changes (inflammation, atrophy, intestinal metaplasia, dysplasia) were blindly assessed using the updated Sydney scoring system (0 = normal to 3 = severe).

### 2.4 Inflammatory cytokine measurement

Serum levels of TNF (Solarbio, Cat:SEKR-0009), MCP1(Solarbio, Cat:SEKR-0024), IL−6(Solarbio, Cat:SEKR-0005), and IL−10(Solarbio, Cat:SEKR-0006) were measured by ELISA following the manufacturer’s protocols.

### 2.5 Protein expression, gene expression, and molecular interaction analyses

The Western blotting & RT-PCR experimental procedure is consistent with our previous study (22, 23). The following antibodies were used: Keap1 (Proteintech, 10503-2-AP, 1:2000), NRF2 (Solarbio, K011897RR, 1:2000), cGAS (HuaBio, HA500023, 1:2000), STING (HuaBio, STING, 1:2000), p−STING (HuaBio, HA723137, 1:1000), TBK1 (HuaBio, HA601045, 1:2000), p−TBK1 (HuaBio, customized, 1:1000), IRF3 (HuaBio, ET1612-14, 1:1000), p−IRF3 (HuaBio, ET1608-22, 1:1000), ZBP1 (HuaBio, ZBP1, 1:2000), and β−actin (Proteintech, 66009-1-IG, 1:40000). densitometry was performed with ImageJ. To evaluate direct binding between AA and STING, molecular docking was performed using AutoDock Vina with human STING structure (PDB: 4EMU) and AA structure from PubChem; the best pose was selected by binding energy. All−atom molecular dynamics (MD) simulations (100 ns) were conducted to assess complex stability via RMSD, RMSF, SASA, hydrogen bonds, and Rg, with visualization in PyMOL.

### 2.6 Cell culture and *in vitro* experiments

Human gastric epithelial GES−1 cells were cultured in RPMI−1640 with 10% FBS, 100 U/mL penicillin, and 100 μg/mL streptomycin at 37 °C in 5% CO_2_. To establish an *in vitro* GPL model, cells were treated with 5 μmol/L MNNG. AA was dissolved in DMSO and applied at final concentrations of 10, 15, or 20 μM. Folic acid was used for positive control (Sigma-Aldrich F7876) at 10 μmol/L (24). For STING−dependent experiments, cells were pre−incubated with the STING agonist cGAMP 10 μg/mL before AA treatment. The cytotoxic effects of AA were evaluated by CCK−8 assay, and the results confirmed that the selected concentrations were non−cytotoxic; detailed data are provided in the **Supplementary Materials 1**.

### 2.7 Cell viability and mitochondrial membrane potential assays

Cell viability was assessed by CCK−8 assay (450 nm). Mitochondrial membrane potential (MMP) was evaluated by JC−1(Solarbio, M8650) staining, and the red/green fluorescence ratio was calculated. (JC-1 monomers: Ex/Em 490/530 nm; aggregates: 525/590 nm).

### 2.8 Surface plasmon resonance

Direct binding affinity between AA and recombinant human STING (TargetMol, USA, TMPJ-01206)was measured using a Biacore system. STING was immobilized on a sensor chip, and increasing AA concentrations (0–100 μM) were injected. Sensorgrams were fitted to a 1:1 Langmuir binding model to calculate the KD.

### 2.9 Statistical analysis

Data are presented as mean ± SD from at least three independent experiments. Multiple group comparisons were performed by one−way ANOVA followed by Tukey’s or Dunnett’s post−hoc test. Two−group comparisons used Student’s t−test. P < 0.05 was considered statistically significant. GraphPad Prism was used for all analyses.

## 3 Results

### 3.1 AA attenuates histopathological damage, systemic inflammation, and physiological deterioration in GPL rats

**Figure 1:**
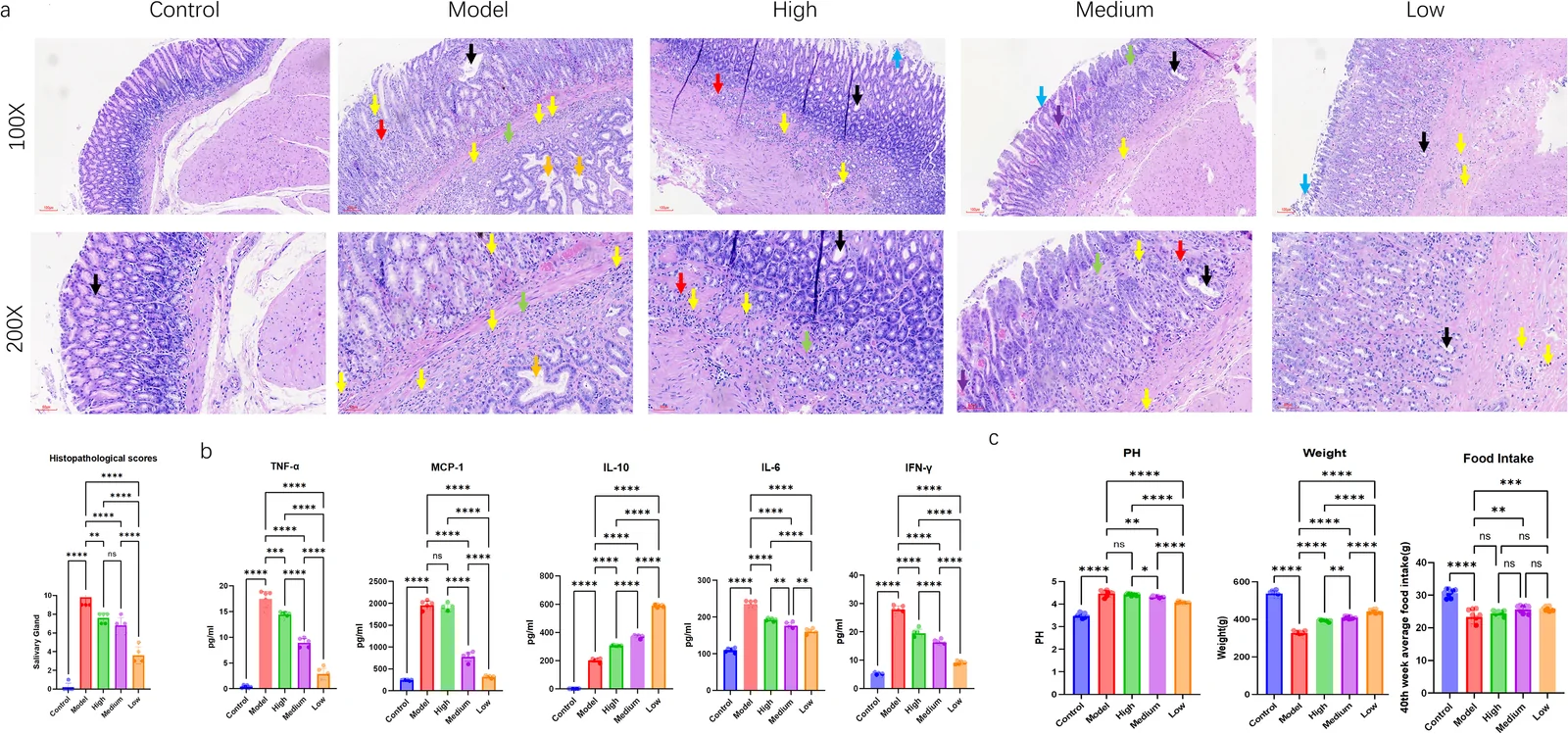
Asiatic acid attenuates histopathological damage, systemic inflammation, and improves physiological parameters in a rat model of gastric precancerous lesions. Groups: Low (12.5 mg/kg AA), Medium (25 mg/kg), and High (50 mg/kg). **(a)** HE staining of gastric tissues for histopathological evaluation (100× and 200× magnifications). **(b)** Serum levels of inflammatory cytokines measured by ELISA. n = 10 per group. **(c)** Physiological parameters including gastric pH, body weight, and average food intake at the 40th week. n = 6 per group. Data are expressed as mean ± SD. ****p < 0.0001, ***p < 0.001, **p < 0.01, *p < 0.05, ns, not significant.

**Figure 1a** presents HE staining of gastric tissues at 100× and 200× magnifications for histopathological assessment. In the control group, HE staining revealed normal morphological structure of epithelial cells in the gastric mucosal layer. The lamina propria contained abundant gastric glands (black arrows), and the muscularis mucosa clearly separated the mucosal layer from the submucosa. Both the muscularis mucosa and the muscularis externa were composed of smooth muscle cells without obvious abnormalities. In the model group, the gastric mucosal epithelial cells were arranged irregularly. A small number of gastric glands in the lamina propria were dilated (black arrows) with eosinophilic substances inside, and rare gastric gland atrophy (red arrows) with reduced volume was observed. The submucosa showed numerous dysplastic glands (orange arrows) with varying sizes and irregular shapes, accompanied by extensive connective tissue hyperplasia (green arrows) and massive infiltration of lymphocytes and granulocytes (yellow arrows). In the high-dose asiatic acid group, a small number of epithelial cells were shed in the gastric mucosal layer (blue arrows). The lamina propria showed frequent gastric gland atrophy (red arrows) with reduced volume, surrounded by extensive connective tissue hyperplasia (green arrows) and mild infiltration of lymphocytes and granulocytes (yellow arrows). Occasional mild dilation of gastric glands (black arrows) was noted. In the medium-dose group, occasional epithelial cell shedding was observed in the gastric mucosal layer (blue arrows). The lamina propria exhibited rare gastric gland atrophy (red arrows) with reduced volume, surrounded by mild connective tissue hyperplasia (green arrows). Occasional gastric gland dilation (black arrows) with eosinophilic substances inside and minor hemorrhage (purple arrows) were detected. Rare infiltration of lymphocytes (yellow arrows) was seen in the lamina propria and submucosa. In the low-dose group, a small number of epithelial cells were shed in the gastric mucosal layer (blue arrows). A few gastric glands in the lamina propria showed mild dilation (black arrows) with eosinophilic substances inside. The submucosa presented small-area edema with loose arrangement of connective tissue, accompanied by mild infiltration of lymphocytes and granulocytes (yellow arrows).

Compared with the normal control group, the model group exhibited significantly elevated levels of all pro-inflammatory cytokines and markedly decreased IL-10 levels (****p < 0.0001). asiatic acid treatment dose-dependently reversed these inflammatory abnormalities, with the low-dose group showing the most pronounced effect, restoring cytokine levels toward normal ranges (**Figure 1b**). **Figure 1c** depicts the physiological parameters, including gastric pH, body weight, and average food intake at the 40th week. Rats in the model group had significantly increased gastric pH (indicating impaired gastric acid secretion), reduced body weight, and decreased food intake compared with controls (****p < 0.0001, ***p < 0.001, **p < 0.01). asiatic acid administration dose-dependently ameliorated these physiological dysfunctions, with the low-dose group demonstrating significant improvements in all three parameters. Collectively, these results demonstrate that asiatic acid effectively mitigates gastric histopathological damage, systemic inflammation and improves general physiological status in rats with gastric precancerous lesions, supporting its potential as a therapeutic agent for this condition.

**Figure 2:**
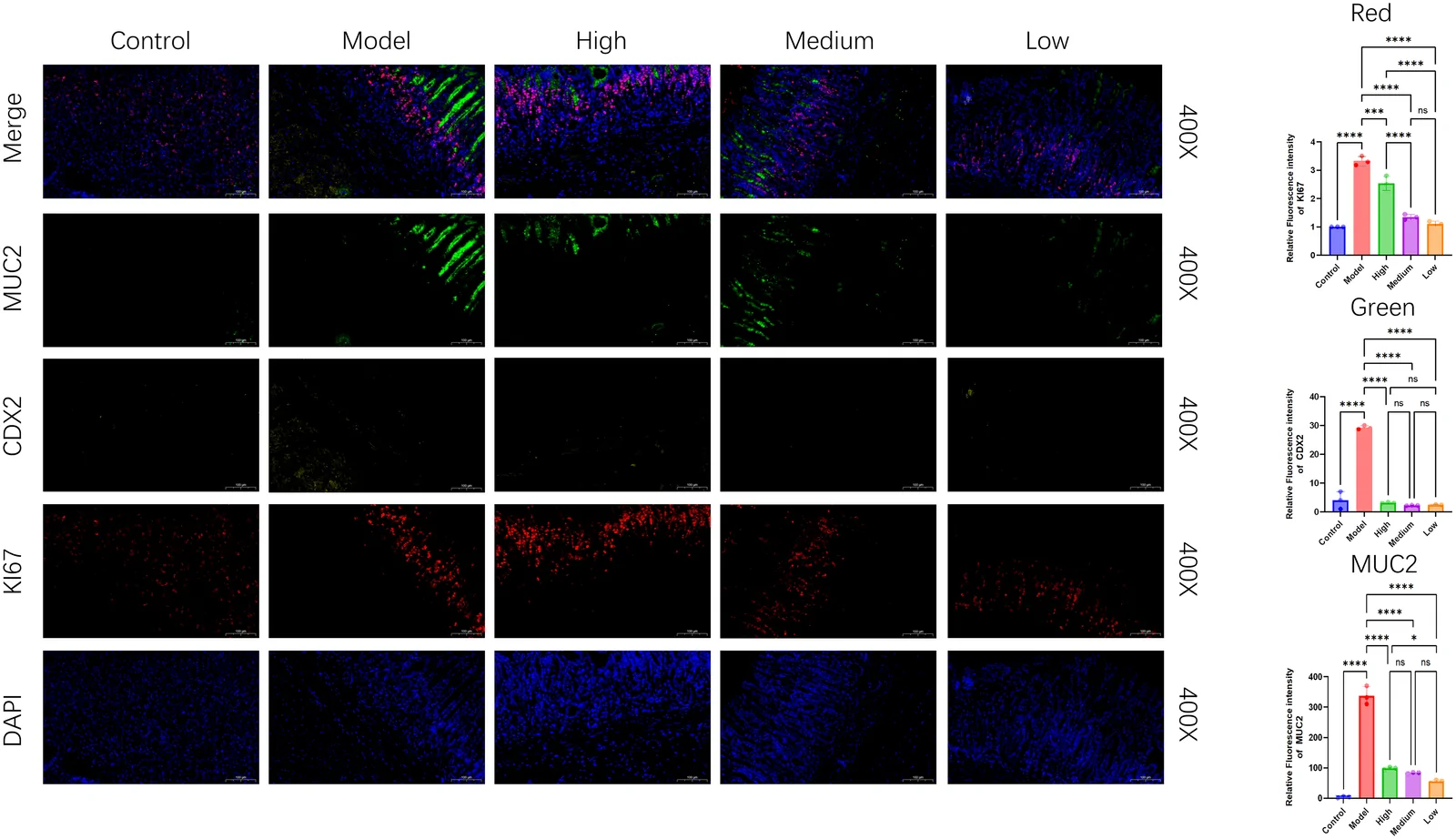
Effects of asiatic acid on intestinal metaplasia markers and cell proliferation in rats with gastric precancerous lesions (immunofluorescence staining). Representative immunofluorescence images (400×, scale bar = 20 μm). Nuclei were stained with DAPI (blue). Ki67 (red) marks proliferating cells; CDX2 (yellow) and MUC2 (green) mark intestinal metaplasia. Groups:Low(12.5 mg/kg AA), Medium(25 mg/kg AA), and High(50 mg/kg AA). n = 3 per group. Data are expressed as mean ± SD. ****p < 0.0001, ***p < 0.001, *p < 0.05, ns, not significant.

To better evaluate the ameliorative effect of asiatic acid on gastric precancerous lesions, we performed immunofluorescence staining for KI67, MUC2, and CDX2. Semi-quantitative analysis showed that all asiatic acid-treated groups, regardless of dose, exhibited a marked reduction in the expression of these markers (**Figure 2**).

### 3.2 AA suppresses aberrant activation of the cGAS-STING signaling pathway in gastric tissues of GPL rats

**Figure 3:**
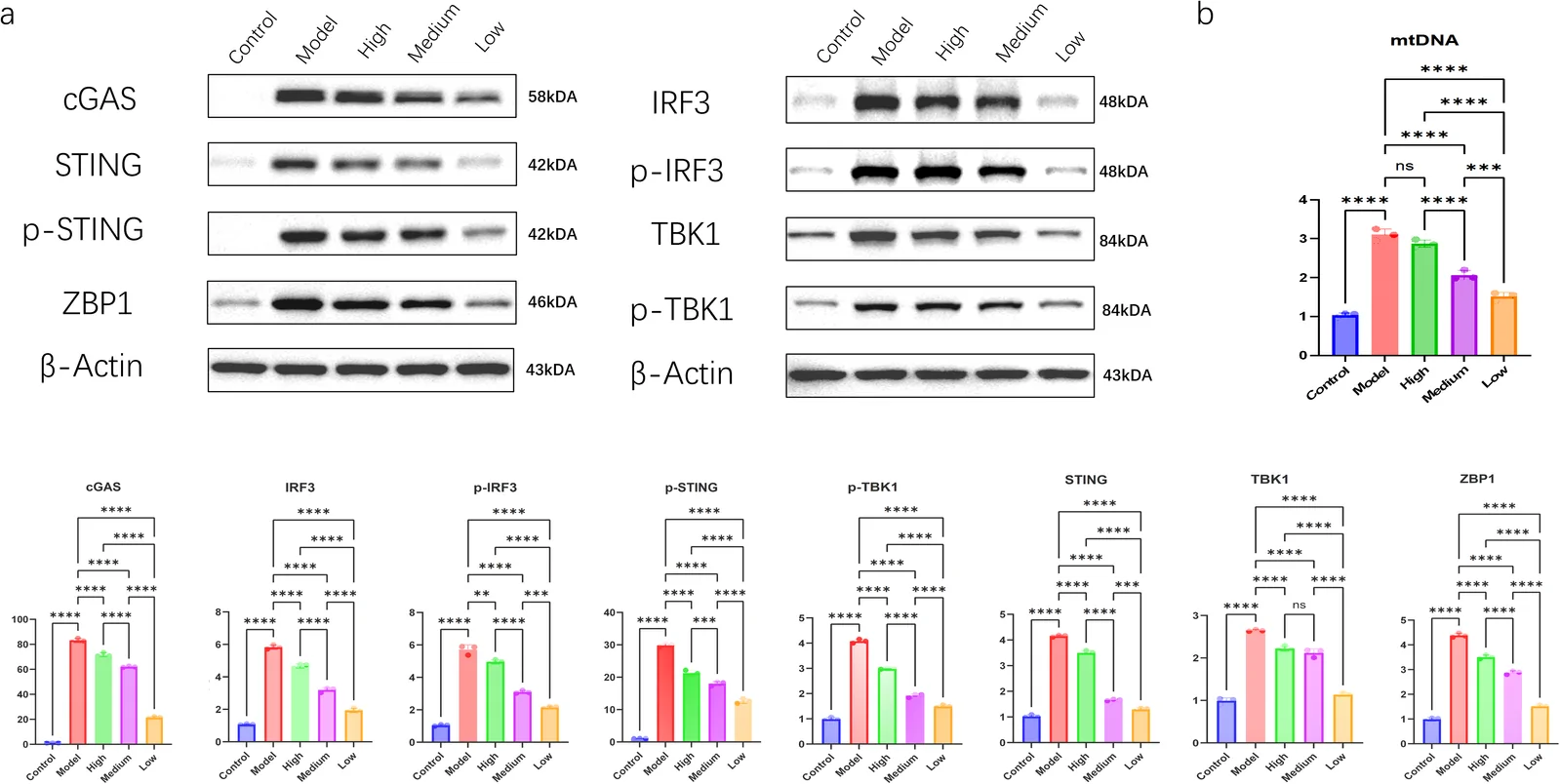
Asiatic acid inhibits abnormal activation of the cGAS-STING signaling pathway in gastric tissues of rats with precancerous lesions. **(a)** Representative Western blot images and quantitative analysis of cGAS-STING pathway-related protein expression in gastric tissues. β-Actin served as the loading control. The detected proteins include cyclic GMP-AMP synthase (cGAS), stimulator of interferon genes (STING), phosphorylated STING (p-STING), Z-DNA binding protein 1 (ZBP1), TANK-binding kinase 1 (TBK1), phosphorylated TBK1 (p-TBK1), interferon regulatory factor 3 (IRF3), and phosphorylated IRF3 (p-IRF3). Data are expressed as mean ± SD. n = 3 per group. ****p < 0.0001, ***p < 0.001, ns: not significant. **(b)** Quantification of mitochondrial DNA (mtDNA) content in gastric tissues Data are shown as mean ± SD (n = 3 per group). ****p < 0.0001, ***p < 0.001, **p < 0.01, ns, not significant.

To explore the molecular mechanism underlying the therapeutic effect of asiatic acid on gastric precancerous lesions, we detected the expression and phosphorylation levels of core proteins in the cGAS-STING innate immune signaling pathway in rat gastric tissues using Western blot (**Figure 3a**).

Compared with the control group, the model group showed a dramatic upregulation in the protein expression of cGAS, STING, p-STING, ZBP1, TBK1, p-TBK1, IRF3, and p-IRF3 (all ****p < 0.0001), indicating robust and excessive activation of the cGAS-STING pathway in gastric precancerous lesions. asiatic acid administration significantly reversed these abnormal changes in a dose-dependent manner. The high-dose asiatic acid group exhibited the most pronounced inhibitory effect, effectively reducing the expression of all detected pathway proteins toward normal levels. Notably, no statistically significant difference was observed in total TBK1 protein expression between the medium-dose asiatic acid group and the model group (ns). Given the role of mtDNA as a key upstream activator of cGAS-STING, we measured mtDNA and found a pronounced increase in the Model group that was dose-dependently attenuated by asiatic acid, corroborating our Western blot findings (**Figure 3b**).

These findings collectively demonstrate that asiatic acid can effectively suppress the aberrant activation of the cGAS-STING signaling pathway in GPL rats, which may represent a key molecular mechanism contributing to its anti-precancerous activity.

### 3.3 AA ameliorates gut microbiota dysbiosis and restores the Nrf2-Keap1 antioxidant axis

**Figure 4:**
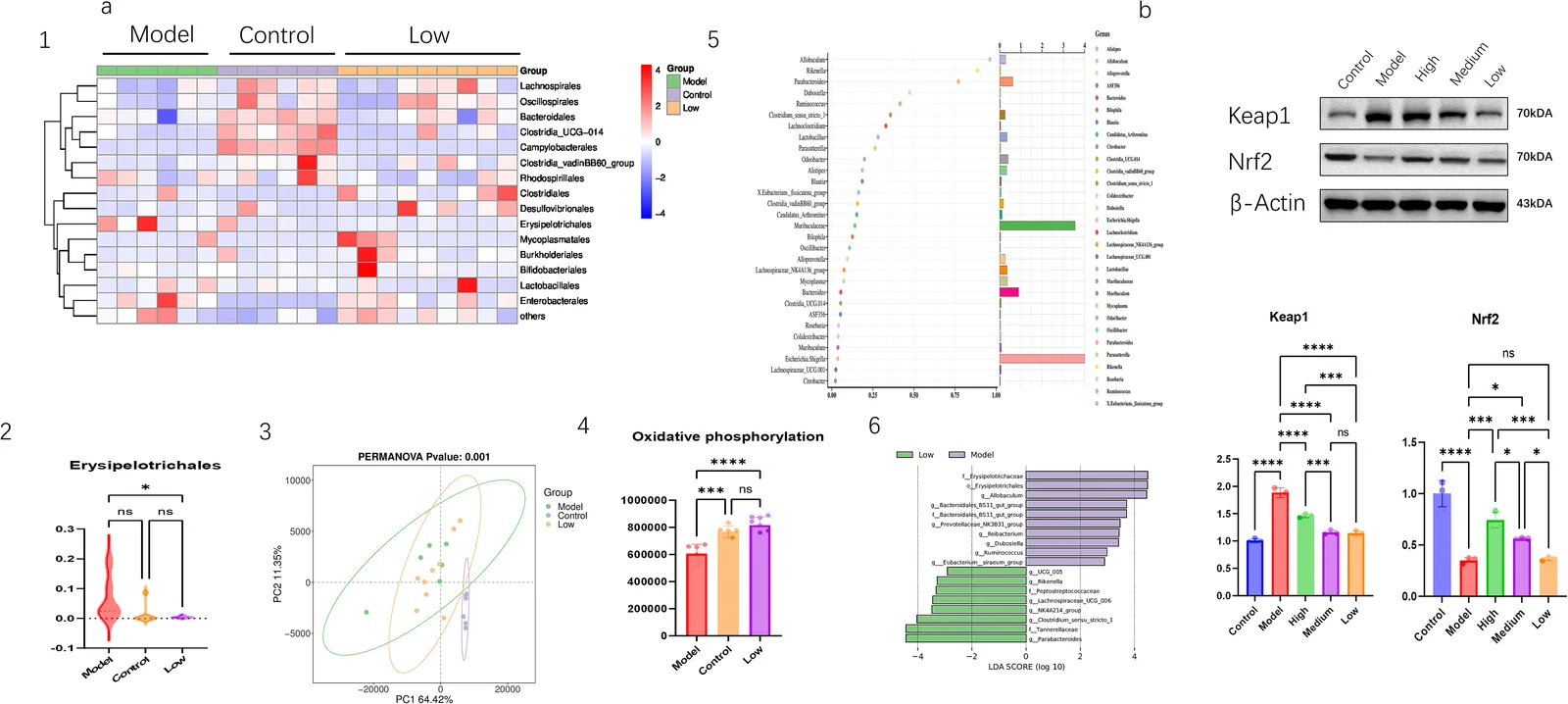
Asiatic acid ameliorates gut microbiota dysbiosis and modulates the Nrf2-Keap1 antioxidant pathway in rats with gastric precancerous lesions **(a)** 16S rRNA gene sequencing analysis of gut microbiota composition and function in rat fecal samples. (a1) Heatmap showing the relative abundance of differential bacterial taxa at the order level between the low-dose asiatic acid group, control group and the model group. (a2) Box plot illustrating the relative abundance of Erysipelotrichales. (a3) Principal coordinate analysis (PCoA) based on Euclidean distances demonstrating the overall structural differences in gut microbiota among groups (PERMANOVA P value = 0.001). (a4) PICRUSt2 functional prediction analysis showing the relative abundance of the oxidative phosphorylation pathway. (a5) Random Forest analysis identifying the most discriminative bacterial taxa between the low-dose asiatic acid group and the model group. (a6) Linear discriminant analysis effect size (LEfSe) analysis showing differentially abundant bacterial taxa with LDA score > 2.0. **(B)** Western blot analysis and quantitative results of Keap1 and Nrf2 protein expression in gastric tissues. β-Actin served as the loading control. Data are expressed as mean ± SD. n = 3 per group. ****p < 0.0001, ***p < 0.001, *p < 0.05, ns, not significant.

To investigate whether the therapeutic effect of asiatic acid on gastric precancerous lesions is associated with the regulation of gut microbiota, we performed 16S rRNA gene sequencing on fecal samples from rats in different groups. (**Figure 4**).

Principal coordinate analysis (PCoA) based on Euclidean distances revealed a clear separation of gut microbiota structure between the Low-dose group and the model group, with the model group distinctly deviating from the control group (PERMANOVA P = 0.001). [**Figure 4a** (3)] At the order level, the model group appeared to show a marked increase in the abundance of potentially pro-inflammatory bacteria such as Erysipelotrichales compared with the control group. Notably, the relative abundance of Erysipelotrichales was significantly elevated in the model group (p < 0.05) [**Figure 4a** (2)], and this change pattern closely paralleled the serum levels of the pro-inflammatory cytokine TNF-α observed in our previous ELISA results. Both Erysipelotrichales and TNF-α were dramatically upregulated in the model group and reduced following asiatic acid treatment, suggesting a potential association between this specific bacterial taxon and systemic inflammation in gastric precancerous lesions.

PICRUSt2 functional prediction analysis indicated that the oxidative phosphorylation pathway was significantly downregulated in the model group compared with the control group (p < 0.01) [**Figure 4a** (4)]. Low-dose asiatic acid treatment effectively restored the abundance of this pathway to near-normal levels (ns vs control, p < 0.0001 vs model). Random forest and LEfSe analyses further identified Erysipelotrichales [**Figure 4a** (5) (6)], Lactobacillus and Clostridium as the key differential bacterial taxa contributing to the separation among the Low-dose asiatic acid group, model group, and control group.

Given the close relationship between oxidative phosphorylation and the Nrf2-Keap1 signaling pathway, we further verified the expression of key proteins in this pathway using Western blot. Compared with the control group, the model group exhibited significantly decreased Nrf2 protein expression and increased Keap1 protein expression (p < 0.0001), suggesting inhibition of the Nrf2-Keap1 antioxidant pathway in gastric precancerous lesions. Compared with the model group, asiatic acid treatment significantly upregulated Nrf2 expression in a dose-dependent manner, with the high-dose and medium-dose groups showing the most pronounced effects (p < 0.01 and p < 0.01, respectively). For Keap1 expression, all asiatic acid treatment groups showed significant downregulation compared with the model group (high-dose: ***p < 0.001; medium-dose: ****p < 0.0001; low-dose: ****p < 0.0001). Notably, the high-dose group had significantly higher Keap1 levels than both the medium-dose and low-dose groups (p < 0.01), while no statistically significant difference was observed between the medium-dose and low-dose groups (ns) (**Figure 4b**).

These results suggest that asiatic acid can ameliorate gut microbiota dysbiosis toward the control group profile and modulate the Nrf2-Keap1 antioxidant pathway, which may contribute to its protective effect against gastric precancerous lesions.

### 3.4 AA protects against MNNG−induced GES−1 cell injury by inhibiting the cGAS−STING pathway and improving mitochondrial function

**Figure 5:**
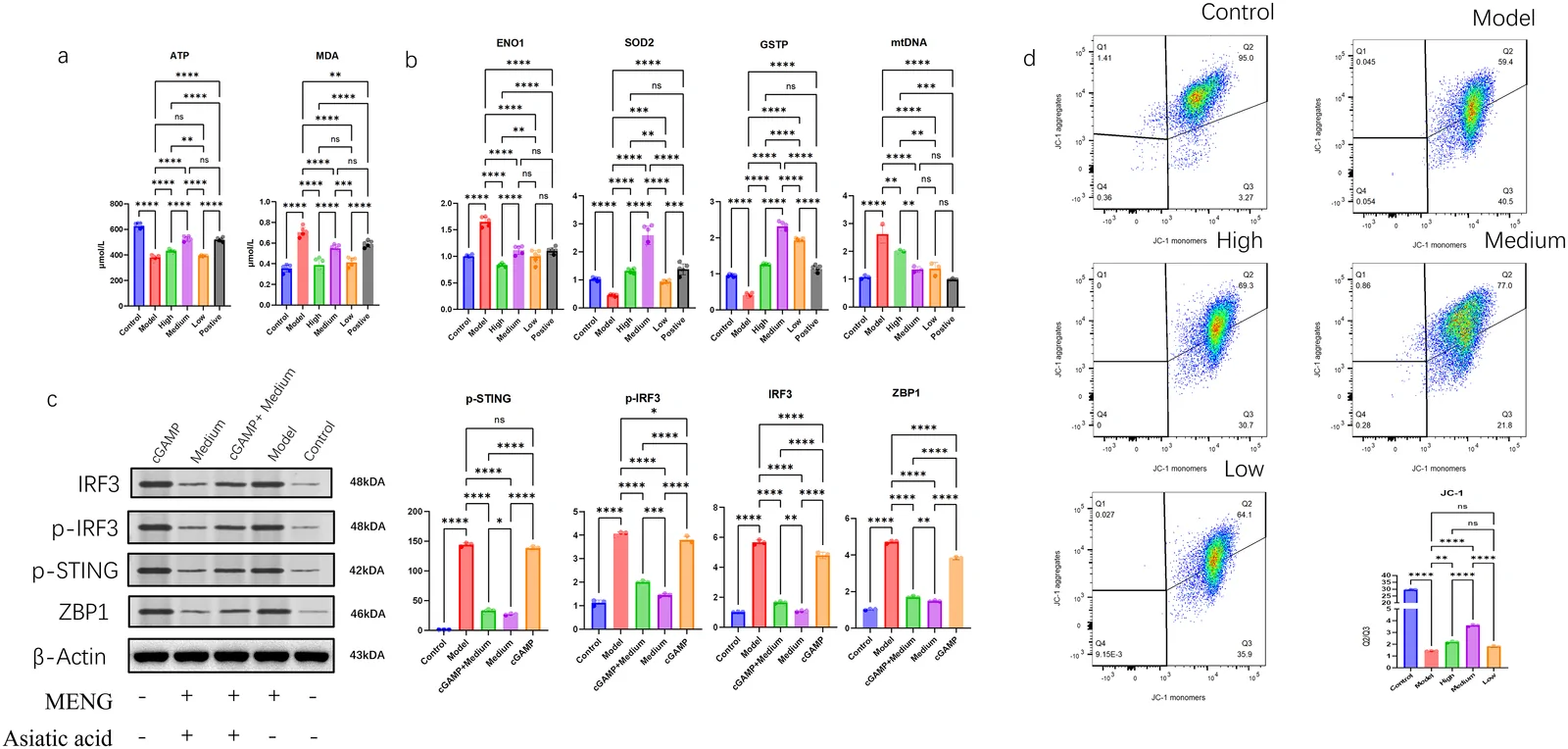
Asiatic acid MNNG-induced GES-1 cell injury by inhibiting the cGAS-STING signaling pathway and improving mitochondrial function. **(a)** ELISA detection of ATP and MDA levels in cell culture supernatants. **(b)** qRT-PCR analysis of the mRNA expression levels of SOD2, ENO1, and GSTP. **(c)** Rescue experiment: Western blot analysis and quantitative results of cGAS-STING pathway-related proteins (IRF3, p-IRF3, p-STING, ZBP1) in GES-1 cells. β-Actin served as the loading control. **(d)** JC-1 flow cytometry analysis of mitochondrial membrane potential (MMP) and quantitative analysis of the Q2/Q3 ratio. Data are expressed as mean ± SD. n = 3 per group. ****p < 0.0001, ***p < 0.001, **p < 0.01, *p < 0.05, ns, not significant.

To further validate the protective effect of asiatic acid at the cellular level and confirm the molecular mechanisms identified in our *in vivo* rat experiments, we established a gastric precancerous lesion cell model by treating human gastric mucosal epithelial GES-1 cells with MNNG. This *in vitro* system allowed us to precisely dissect the direct effects of asiatic acid on gastric epithelial cells independent of systemic factors (**Figure 5**).

First, we examined the effects of asiatic acid on energy metabolism and oxidative stress in MNNG-injured GES-1 cells. ELISA results showed that compared with the control group, the model group exhibited significantly decreased ATP levels and increased MDA levels (****p < 0.0001), (**Figure 5a**) indicating impaired energy metabolism and enhanced lipid peroxidation. These findings are consistent with our *in vivo* observations of mitochondrial dysfunction and oxidative damage in gastric tissues of rats with precancerous lesions. asiatic acid treatment dose-dependently reversed these changes, with the high-dose and medium-dose groups showing significant improvements in both ATP and MDA levels (p < 0.01). qRT-PCR analysis revealed that MNNG treatment significantly downregulated the mRNA expression of mtDNA, and antioxidant enzymes SOD2, GSTP, while upregulating the glycolytic enzyme ENO1 (****p < 0.0001) (**Figure 5b**) This shifts from oxidative phosphorylation to glycolysis mirrors the metabolic reprogramming observed in our *in vivo* PICRUSt2 analysis, which showed downregulation of the oxidative phosphorylation pathway in the model group. asiatic acid administration effectively restored the expression of these genes toward normal levels.

Subsequently, we evaluated mitochondrial function using JC-1 staining. Flow cytometry results demonstrated that MNNG treatment caused a significant decrease in mitochondrial membrane potential, as evidenced by the reduced Q2/Q3 ratio in the model group compared with the control group (****p < 0.0001). Mitochondrial dysfunction is a well-known trigger for the release of mitochondrial DNA into the cytoplasm, which is the primary activator of the cGAS-STING pathway that we found to be abnormally activated in our *in vivo* rat studies. asiatic acid treatment dose-dependently increased the Q2/Q3 ratio, indicating improved mitochondrial function and reduced potential for cGAS-STING pathway activation.

To confirm whether the protective effect of asiatic acid is mediated through the inhibition of the cGAS-STING signaling pathway, we performed a rescue experiment using cGAMP, a specific agonist of the cGAS-STING pathway. Western blot results showed that MNNG treatment significantly upregulated the protein expression of p-STING, p-IRF3, IRF3, and ZBP1 (****p < 0.0001), which was fully consistent with our *in vivo* findings in rat gastric tissues. Medium-dose asiatic acid treatment significantly inhibited the activation of these pathway proteins. However, co-treatment with cGAMP partially reversed the inhibitory effect of asiatic acid on the cGAS-STING pathway, as evidenced by the increased expression of p-STING, p-IRF3, IRF3, and ZBP1 compared with the asiatic acid alone group. This rescue effect provides direct causal evidence that asiatic acid exerts its protective effects primarily through inhibiting the cGAS-STING signaling pathway, confirming the mechanistic hypothesis derived from our *in vivo* experiments.

Collectively, these *in vitro* results demonstrate that asiatic acid can alleviate MNNG-induced GES-1 cell injury by improving mitochondrial function, reducing oxidative stress, and inhibiting the cGAS-STING signaling pathway. The remarkable consistency between our *in vitro* and *in vivo* findings supports the translational potential of asiatic acid as a novel therapeutic agent for gastric precancerous lesions.

### 3.5 AA directly binds to STING protein as revealed by molecular docking, MD simulations, and SPR

**Figure 6:**
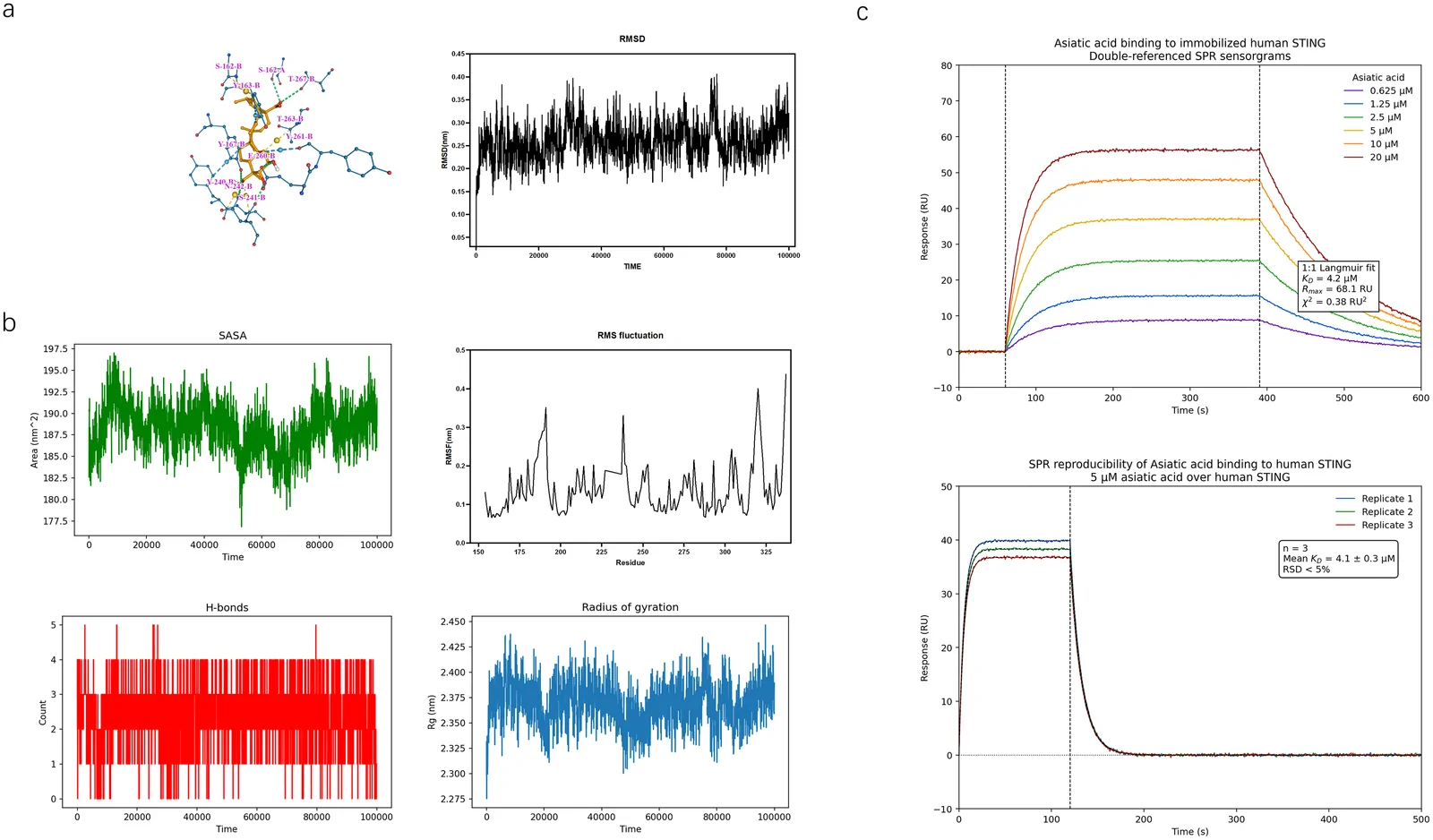
Computational and biophysical evaluation of the interaction between asiatic acid and STING protein. **(a)** Molecular docking visualization of asiatic acid interaction with the STING protein. **(b)** Molecular dynamics (MD) simulation analysis of the asiatic acid-STING complex 100 ns. **(c)** Surface plasmon resonance (SPR) analysis of the interaction between asiatic acid and STING.

To elucidate the direct molecular interaction between asiatic acid and its putative target STING protein, we performed integrated computational and experimental binding studies (**Figure 6**).

Computational chemistry approaches, such as molecular docking and molecular dynamics simulations, have had a huge impact on pharmacological research and are playing an increasingly important role (25, 26).

Molecular docking analysis revealed that asiatic acid could stably bind to the ligand-binding domain of STING with a high binding affinity of -9.7 kcal/mol. The detailed interaction analysis showed that asiatic acid formed multiple hydrogen bonds and hydrophobic interactions with key amino acid residues in the STING binding pocket, including Phe210, Ser213, Tyr240, Val239 and Tyr242, which are critical for STING activation.

To further validate the dynamic stability of the asiatic acid-STING complex, we conducted 100 ns all-atom molecular dynamics simulations. The root means square deviation (RMSD) of the protein backbone remained relatively stable throughout the simulation period, indicating that the complex maintained a stable conformation. The root means square fluctuation (RMSF) analysis showed that the residues in the binding pocket exhibited low flexibility, suggesting that asiatic acid binding restricted the conformational changes of these regions. Additionally, the solvent-accessible surface area (SASA), number of hydrogen bonds, and radius of gyration (Rg) all remained stable during the simulation, further confirming the structural stability of the complex and the persistent interaction between asiatic acid and STING.

Finally, we used surface plasmon resonance (SPR) technology to directly measure the physical interaction between asiatic acid and recombinant STING protein *in vitro*. The sensorgrams showed a concentration-dependent increase in binding response, and the steady-state affinity analysis yielded a dissociation constant (KD) of 4.0 μM, indicating a moderate but specific binding affinity between asiatic acid and STING. The residual plot demonstrated a good fit of the experimental data to the 1:1 binding model, confirming the specificity of the interaction.

These results provide direct evidence that asiatic acid can specifically bind to the STING protein with high affinity and stabilize its conformation, which is consistent with our previous *in vivo* and *in vitro* findings showing that asiatic acid inhibits the activation of the cGAS-STING signaling pathway. This direct binding interaction represents the molecular basis for the regulatory effect of asiatic acid on the cGAS-STING pathway.

## 4 Discussion

In this study, we demonstrated through molecular docking, 100 ns molecular dynamics simulations, and surface plasmon resonance (SPR, KD = 4.0 μ M) that AA can bind to STING protein. *In vitro* and *in vivo* experiments, we observed that AA can reduce the expression of p-STING, p-TBK1, and p-IRF3. The cGAMP rescue experiment further supports the action of AA upstream of STING. These findings position AA as a regulatory factor in the STING pathway. It should be pointed out that the role of STING in cancer is not fixed, but depends on its activation mode: acute activation helps enhance anti-tumor immunity, while chronic activation promotes disease progression through sustained inflammatory response. The gastric precancerous lesions (GPL) model used in this study accurately represents the latter - a persistent low-grade inflammatory state, therefore the inhibition of STING by AA is beneficial in this specific situation.

In the GPL rat model, we also observed dysbiosis of the gut microbiota, and this observation is in agreement with findings reported in other publications (27), and AA was able to dose dependently reverse this dysbiosis and regulate the Nrf2/Keap1 antioxidant axis (Nrf2 upregulated, Keap1 downregulated). It should be pointed out that the causal relationship between microbial dysbiosis, oxidative stress, and STING activation has not been directly confirmed through experiments such as fecal microbiota transplantation or sterile animals. Therefore, we consider it as a correlation-based discovery rather than a confirmed mechanism loop. In addition, this study found that low-dose AA had similar or even slightly better effects on certain inflammatory indicators than medium to high doses. This non monotonic dose-response has been reported in phytochemicals (such as toxic excitatory effects), but core pathological indicators and mitochondrial function related indicators still showed clear dose-dependent improvements. AA simultaneously inhibits STING and activates Nrf2, but cGAMP rescue experiments can only partially reverse the upregulation of Nrf2, indicating that Nrf2 activation may be partially independent of STING inhibition, which is consistent with AA’s inherent antioxidant activity. In summary, AA improves the local microenvironment before gastric cancer through multi-target effects (anti-inflammatory, antioxidant, regulating gut microbiota, inhibiting STING pathway), providing experimental evidence for its development as a candidate drug for chemoprevention.

Recently, extensive evidence has established a functional crosstalk between STING and the Keap1-Nrf2 antioxidant axis. On one hand, STING activation promotes Nrf2 proteasomal degradation through the OLA1-Keap1 pathway, thereby suppressing the endogenous antioxidant defense (28). On the other hand, Nrf2 acts as a negative regulator of STING by reducing STING mRNA stability, thus dampening STING-mediated inflammatory signaling (29). These reciprocal regulatory loops constitute an integrated KEAP1-NRF2-STING signaling axis, in which the two pathways mutually constrain each other to fine-tune the cellular redox balance and innate immune activation.

Of course, this study also has certain limitations: the binding site between AA and STING has not been validated through site directed mutagenesis or co crystallization; Lack of STING gene knockout model to confirm pathway specificity; No positive control (such as folic acid) was set up in animal experiments; Long term tumor incidence rate was not evaluated during the 8-week treatment. The above limitations have not weakened the value of AA as a lead compound, but instead provide a clear direction for its further optimization and validation. AA not only effectively inhibits the chronic activation of the STING pathway, but also has anti-inflammatory, antioxidant, and regulatory effects on gut microbiota, demonstrating a unique advantage of multi-target intervention in precancerous lesions of gastric cancer.

## 5 Conclusion

In summary, this study demonstrates that AA suppresses the cGAS-STING pathway, thereby improving the gastric microenvironment in GPL by restoring Nrf2−Keap1 antioxidant signaling and improving mitochondrial function. These findings provide a mechanistic rationale for developing AA as a novel chemopreventive agent targeting the cGAS-STING pathway in gastric carcinogenesis.

## Data Availability

The original contributions presented in the study are included in the article/**Supplementary Material**. Further inquiries can be directed to the corresponding authors.
