# Supplementary material for: Asiatic acid remodels the gastric precancerous immune microenvironment by targeting STING
Source: Front Immunol. 2026 Aug 20;17:1896372. doi: 10.3389/fimmu.2026.1896372 (PMC13538884; doi:10.3389/fimmu.2026.1896372)
Supplement: Supplementary file 1 [file DataSheet1.pdf]

Supplementary Material

1.1 Supplementary Figures

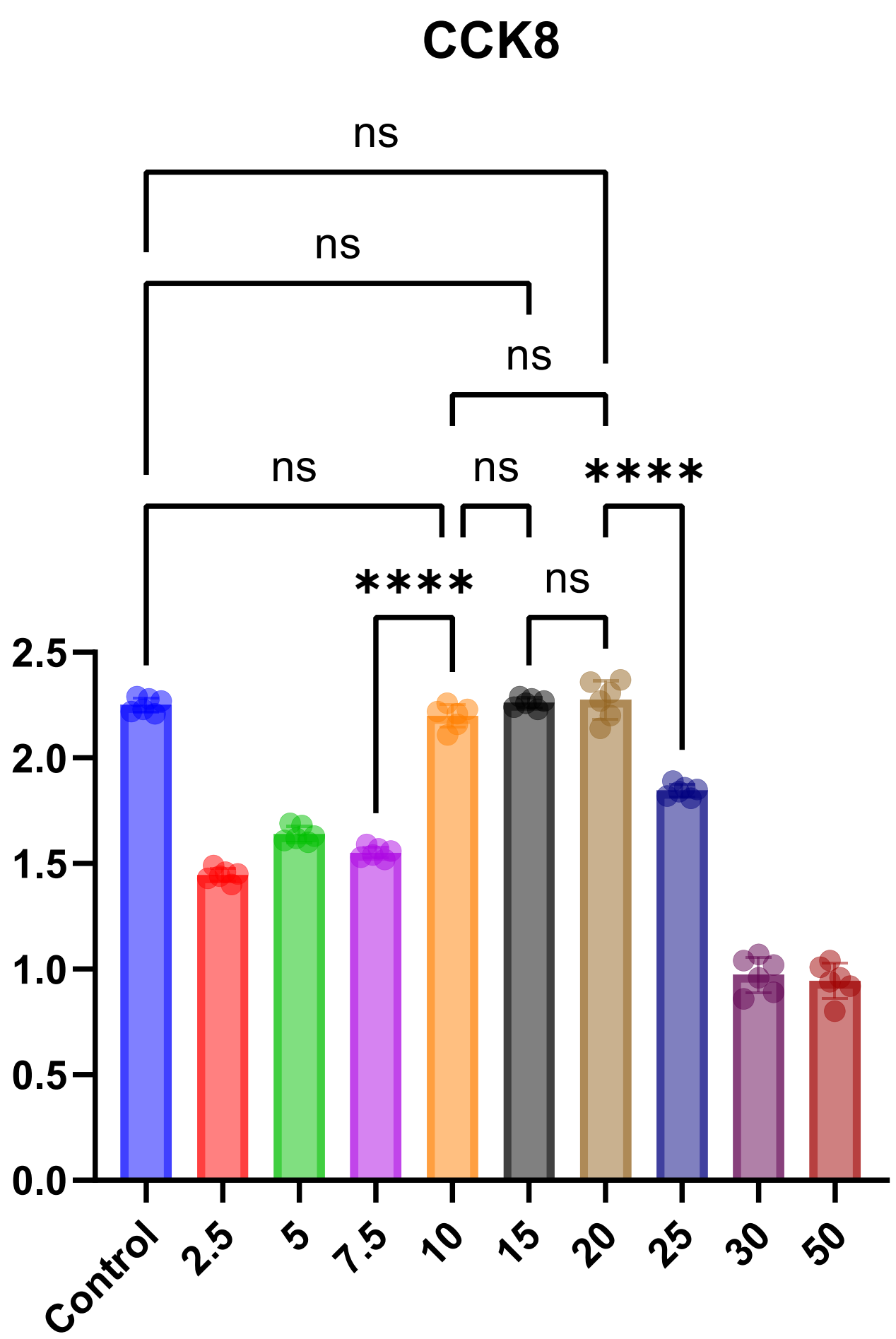

Supplementary Figure 1. Effects of different concentrations of asiatic acid on the viability of GSE-1 cells.

Cell viability was measured by CCK-8 assay after 24 h treatment. Data are presented as mean ± SD. n = 6 per group. *p < 0.05, **p < 0.01 vs. control group; ns, not significant. Concentrations of 10, 15, and 20 μM were selected for subsequent experiments based on non-cytotoxic and effective conditions..
